# Persistent Immune Activation in CVID and the Role of IVIg in Its Suppression

**DOI:** 10.3389/fimmu.2014.00637

**Published:** 2014-12-16

**Authors:** Dominic Paquin-Proulx, Johan K. Sandberg

**Affiliations:** ^1^Department of Medicine, Center for Infectious Medicine, Karolinska Institutet, Karolinska University Hospital, Stockholm, Sweden

**Keywords:** CVID, IVIg, iNKT cells, CD8 T cells, CD4 T cells, dendritic cells, immune activation, IgG replacement therapy

## Abstract

Common variable immunodeficiency (CVID) is one of the most common and clinically important primary immune deficiencies. CVID patients have poor humoral immunity, resulting in recurrent infections of the gastrointestinal and upper respiratory tracts, as well as increased incidence of some forms of cancers and autoimmune diseases. The treatment for CVID is IgG replacement, often given as intravenous immunoglobulins (IVIg). IVIg consists of monomeric IgG purified from pooled plasma from healthy donors and is used to treat an increasing number of conditions including autoimmune diseases. In the case of CVID, IVIg has mainly been seen as reconstitution therapy, providing patients with pathogen-specific antibodies. Recent evidence shows that IVIg has diverse effects on the immune system of CVID patients, and one important component is that IVIg alleviates the state of chronic immune activation. In this review, we will discuss causes and consequences of persistent immune activation in CVID, possible underlying mechanisms for how IVIg treatment reduces immune activation, and implications for our understanding of primary as well as acquired immune deficiencies.

## Introduction

Diagnosis of common variable immunodeficiency (CVID) is based on low levels of IgG and IgA and lack of specific IgG response following vaccination (1). Several genetic mutations associated with CVID have been identified but for a significant proportion of patients the exact cause is unknown (2). CVID patients, thus, are a heterogeneous group characterized by poor humoral immunity, resulting in recurrent infection of the gastrointestinal and upper respiratory tracts. CVID is also associated with non-infection related complications including cancers, mainly non-Hodgkin’s lymphoma, and autoimmune diseases such as idiopathic thrombocytopenia purpura and autoimmune hemolytic anemia, contributing to a significantly shorter life expectancy (3, 4). It has become clear that defects in the immune system in CVID go beyond humoral immunity with significant changes and persistent activation of the cellular immune system, involving dendritic cells (DCs), CD8 T cells, CD4 T cells, invariant natural killer T (iNKT) cells, and regulatory T cells (Tregs). The treatment for CVID is IgG replacement, often given as intravenous immunoglobulins (IVIg). IVIg consists of monomeric IgG purified from pooled plasma from healthy donors and it is used to treat an increasing number of conditions (5), including autoimmune diseases. The mechanisms of action of IVIg in treatment of autoimmune diseases are numerous and controversial (6, 7). In the case of CVID, it has mainly been seen as a reconstitution therapy, providing patients with pathogen-specific antibodies, but evidence now shows that IVIg has a variety of effects on the immune system of CVID patients (8) and can alleviate the state of chronic immune activation. In this review, we will discuss the causes and consequences of innate and adaptive immune activation, and how IVIg treatment reduces immune activation in CVID focusing on data obtained *ex vivo*. We will also discuss the similarities between primary and secondary immune deficiencies and the possible implications for our understanding of those diseases.

## Innate Immune Activation

Common variable immunodeficiency patients present defects in several arms of the innate immune system. Natural killer (NK) cells express a wide repertoire of activating and inhibitory receptors and are part of the innate defenses against viral infections and tumors (9). NK cells were first recognized for their cytotoxic capacity but they can also produce cytokines and have regulatory properties (10). The frequency of NK cells has been reported to be lower in CVID (11). Detailed studies of NK cells in CVID are lacking, and the effects IVIg treatment may have on NK cell frequency, phenotype, and functions are unknown. Because of their antitumor function, decline of NK cells could contribute to the increased risk of cancer in CVID patients.

Polymorphonuclear neutrophils (PMNs) are an important component of the innate immune system. In response to pathogens, PMN can rapidly migrate to the site of inflammation and have microbicidal activity by the release of proteolytic enzymes and antimicrobial peptides as well as production of reactive oxygen species (ROS) (12). In CVID, PMNs have been reported to express lower levels of CD11b, CD16b, and CD15, suggesting a maturation defect (13). The same study also reported impaired phagocytosis of *E. coli* and reduced ROS production after TLR stimulation by PMN from CVID patients. The patients in this study were all under IVIg treatment, and the effects of IVIg on PMN phenotype and function therefore remain undetermined. However, *in vitro* experiments performed on whole blood from healthy individuals suggest that low doses of IVIg, as used for treatment of CVID, can induce CD11b expression and increase the ROS response (14).

Monocytes are myeloid-derived cells with phagocytosis and antigen presentation capacities. They can rapidly differentiate into tissue-resident macrophages and DCs after leaving the blood stream. Monocytes play an important role in various inflammatory conditions (15). In CVID, the frequency of pro-inflammatory CD14^bright^ CD16^+^ monocytes is elevated and these cells express higher levels of HLA-DR indicating a higher activation level (16). Another study showed that IVIg temporarily reduced the frequency of pro-inflammatory monocytes 4 h after injection and that the levels returned to baseline after 20 h (17). Furthermore, IVIg may reduce TNF production by monocytes from CVID patients, possibly by triggering of the inhibitory receptor FcγRIIb (17). Monocytes from CVID patients were also found to have increased production of ROS, and this was inversely correlated with CD4 counts (18).

Dendritic cells are professional antigen presenting cells (APCs) specialized in capturing, processing, and presenting antigens to initiate immune responses to pathogens. After TLR activation, DCs will mature and increase the expression of co-stimulatory molecules to provide the second signal needed to activate T cells. Bayry et al. showed that *in vitro* differentiation of monocytes from CVID patients into DCs (19) is defective, and that normal differentiation could be restored by natural antibodies against CD40 present in IVIg (20). However, the *in vivo* relevance of this mechanism remains to be investigated as *ex vivo* DCs from CVID patients present a different phenotype. CVID patients have reduced frequencies of plasmacytoid and myeloid DCs (21), and the residual myeloid DCs have increased expression of co-stimulatory molecules CD80 and CD83 (22). The frequency of myeloid DCs is partially restored following initiation of IVIg treatment and the expression of CD80 is significantly decreased. Moreover, myeloid DCs in treatment-naïve CVID patients display an abnormal profile of group I CD1 molecules characterized by an elevated representation of the CD1c^+^ subset. In addition, the CD1c^+^ and the CD1c^−^ subsets of DCs have higher CD1a and CD1b expression in these patients (23), whereas CD1d is expressed at similar levels between CVID patients and controls, being present on the majority of the cells. Following the increase in IgG level after initiation of replacement therapy, the CD1c subset frequency is normalized together with the expression levels of CD1a and CD1b, while CD1d expression is unaffected. These findings suggest that IgG can regulate the expression of group I CD1 molecules *in vivo*. Earlier studies *in vitro* indicated that this effect is mediated by binding to the FcγRIIb (24). It remains to be investigated if the increased expression of CD1a in treatment-naïve CVID patients can lead to aberrant activation of the CD1a restricted T cells that are present in the normal repertoire (25).

Because they can rapidly be activated and produce cytokines without previous encounter of their antigen, iNKT cells are sometimes considered to be part of the innate immune system (26). They recognize endogenous and bacterial-derived glycolipids presented by CD1d molecules. It is believed that iNKT cells are important for the control of both bacterial and viral infections and they are also believed to be involved in immune surveillance against cancer and to have the capacity to regulate auto-immunity (26). iNKT cells are numerically reduced in CVID (27) and present elevated expression of HLA-DR, CD161, and PD-1 (22), signs of activation and exhaustion. In addition, the distribution of iNKT cell subsets defined by CD4 and CD8 is skewed in CVID, with an increase in the CD4^+^ and a decrease in the CD8^+^ subset reported in one cohort (28). The function of iNKT seems to be relatively preserved in treatment-naïve CVID patients, as only a trend for reduced IFNγ production was seen after stimulation with the model antigen α-Gal–Cer (29). Increased IFNγ production by iNKT cells was reported for a small number of CVID patients on going IVIg after *in vitro* expansion (30). The frequency of iNKT cells does not improve by reconstitution therapy, and HLA-DR remains elevated. However, expression of CD161 and PD-1 is reduced when CVID patients are under IVIg treatment (22), indicating that IVIg can alleviate iNKT cell activation and exhaustion in patients. Because of the important role of iNKT cells in tumor surveillance and immune regulation, it is possible that the loss of these cells is contributing to the increased risks of cancer and auto-immunity in CVID patients.

## Adaptive Immune Activation

Treatment-naïve CVID patients present low-CD4 T cell counts, in some cases down to numbers that would be considered AIDS-defining in HIV-1 infected patients. Following IVIg initiation, CD4 counts increase in the majority of CVID patients and can reach normal levels in some cases (22, 31). It is noteworthy that a similar effect has been reported in HIV-1 patients treated with IVIg (32, 33). The mechanisms by which IVIg can normalize CD4 counts remain elusive, but Dolcino et al. reported that lower expression of LEPR, a gene important for CD4 proliferation, was normalized after IVIg treatment in CVID patients (31). CD8 T cell counts in CVID patients are in general not different from healthy controls but some patients have an expansion of this population. Therefore, the inverted CD4:CD8 ratio seen in CVID is mostly due to their low-CD4 count. CD4 T cells in CVID have elevated levels of the activation markers Ki67, CD38, and HLA-DR, as well as exhaustion markers PD-1 and CTLA-4. The expression levels of activation and exhaustion markers remained elevated for up to 1 year on IVIg treatment (22). Interestingly, another study found that IVIg treatment could reduce PD-1 expression on CD4 T cells and restore their response to bacteria (34).

Similar to the CD4 T cell compartment, CD8 T cells in treatment-naïve CVID have elevated expression of activation markers Ki67 and co-expression of CD38 and HLA-DR. IgG replacement therapy leads to reduced expression of Ki67, CD38, and HLA-DR on CD8 T cells (22), indicating that IgG replacement may help control infections or infection-associated factors that are implicated in chronic activation of the CD8 T cells. Expression of some activation markers on CD8 T cells and exhaustion markers on CD4 T cells correlate positively with age in IVIg-naïve CVID patients (22), suggesting that immune activation and exhaustion are developing progressively over time. Furthermore, the ratio of activated T cells to Tregs was found to be higher in CVID patients with auto-immunity compared to patients without auto-immunity (35). Therefore, early initiation of IgG replacement therapy in CVID patients may be beneficial by preventing further increase in T cell activation. However, diagnosis of CVID is frequently delayed by 6–8 years after the onset of symptoms (1).

Regulatory T cells are key regulators of immune responses and they play a crucial role in limiting unwanted and persistent immune activation. Several studies demonstrated that Tregs are reduced in CVID patients (22, 36–38) and that residual Tregs appear to have reduced suppressive capacity (39). An increase in Tregs was reported 30 min after IVIg infusion in CVID patients (38). This increase seems to be only transient as no sustained effect on Treg frequency was observed between samples obtained at baseline and up to 1 year after initiation of IVIg treatment (22). The inability of IVIg to restore normal frequency and function of Tregs may contribute to the increased risk of auto-immunity in CVID patients (36).

## What is Driving Immune Activation in CVID?

**Figure 1:**
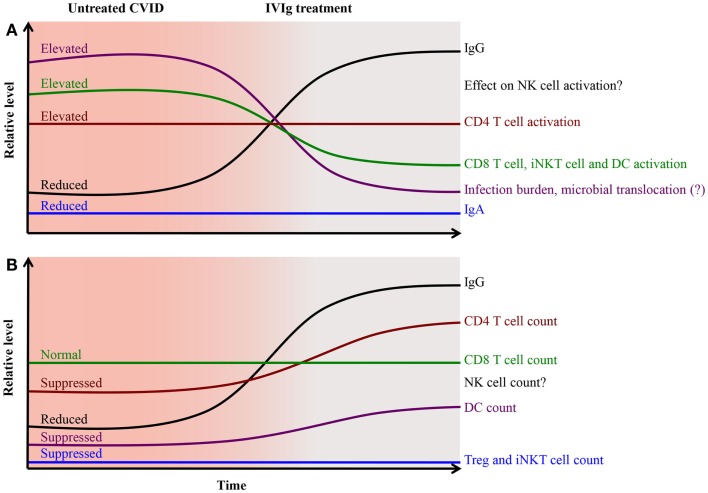
**Pathological changes and activation of cellular immunity in CVID is partially alleviated after immunoglobulin replacement therapy**. IgG replacement therapy restores humoral immunity and provides better control of microbes and pathogens, reducing the infection burden on the immune system. This together with triggering of the FcR-mediated inhibitory effects on antigen presenting cells leads to reduced activation of CD8 T cells, iNKT cells, and DCs **(A)** and improvement in CD4 T cell counts and DC count **(B)**.

Signs of activation in both monocytes and DCs are associated with T cell activation (16, 22, 40), suggesting that persistent innate activation contribute to chronic activation of the adaptive immune system. We propose a model where recurrent and chronic infections at mucosal surfaces in treatment-naïve CVID patients result in sustained activation of monocytes and DCs, and that these cells in turn promote T cell activation (Figure 1). In this model, one possible mechanism by which IVIg reduces activation of T cells is by acting at the level of the APCs. *In vivo* and *in vitro* studies support the notion that IVIg reduces T cells activation indirectly by acting on APCs rather than on the T cells themselves (41–45). IVIg may act directly on APCs via Fc-receptors, or have indirect effects on APCs activation by reducing the infection burden. However, persistent activation of monocytes and DCs is seen in CVID patients even after IVIg therapy and the causes are largely unknown.

CMV infection has been associated with complications in CVID patients (46, 47) and thus CMV is a possible candidate as a cause for chronic immune activation in CVID. Resurgence of endogenous retroviruses (ERVs) in the absence of LPS-specific antibodies has been reported in a mouse model (48). IVIg preparations contain antibodies specific for both CMV (49) and LPS (50), and may therefore help reduce the pressure on the immune system by supporting immune control of CMV and by preventing activation of ERVs. Enteric virus infections were found in 25% of CVID and CVID-like patients compared to 9% in controls, and these infections were associated with increased levels of calprotectin, a marker of inflammation, and low levels of IgA (51). Therefore, enteric viruses may also contribute to increased immune activation in CVID patients. However, only few CVID patients present the CD8 T cell expansion typically associated with chronic viral infections, suggesting that other causes may be involved. Soluble CD14 (sCD14), a marker associated with monocyte activation and possibly microbial translocation, is elevated in CVID (16, 22, 52). LPS levels were found to be elevated in one cohort of treatment-naïve CVID patients and were reduced following replacement therapy (34). However, we and others were unable to detect elevated LPS levels in the circulation of CVID patients [our unpublished data and Ref. (16, 52)]. Therefore, more investigations are needed to clarify the role of microbial translocation in CVID patients. The loss of Tregs as well as iNKT cells with regulatory capacities may contribute to the persistent immune action seen in CVID. These two populations appear to not recover after initiation of IVIg therapy (22), possibly providing an explanation as to why immune activation remains elevated.

## Similarities between Primary and Secondary Immune Deficiencies

Some of the immunological perturbations observed in treatment-naïve CVID patients are strikingly similar to those seen in untreated HIV-1 infection. HIV-1 infection leads to chronic immune activation characterized by increased T cell activation and exhaustion, elevated levels of sCD14 and a partial loss of CD4 T cells, DCs, iNKT cells, and Tregs. These features are also found in CVID patients. T cell activation is closely associated with HIV-1 disease progression (53) but it is unknown if similar associations exists in CVID. IL-6 is a predictor of all-cause mortality (54) and disease progression (55) in HIV-1 infection, and has been associated with opportunistic infections (56) and increased risk of cancer (57). Interestingly, several studies reported an increase in IL-6 in CVID (58); however, it has not been studied as a biomarker of disease progression or complications. CVID and HIV-1 also present a similar signature in gene expression in the intestinal epithelium with up-regulation of innate immune gene and down-regulation of lipid and carbohydrate metabolism genes and transport of micronutrients genes (59), suggesting that events at the mucosal barrier may be involved in the similarities between the two diseases. Moreover, sCD14 levels have been found to associate with immune activation in both CVID (52) and HIV-1 (60), suggesting that monocyte activation is common to both conditions. IL-2 administration in combination with antiretroviral treatment results in increased CD4 counts (61), and expansion of iNKT cells (62) and NK cells (63) in HIV-1 infected patients. IL-2 has also been studied as a complementary therapy in CVID, resulting in increased T cell responses to mitogens and soluble antigens in the absence of changes in CD4 count and NK cell frequency (64) while iNKT cell frequency was not evaluated. Because of the similarities between CVID and HIV-1 disease, it is not unexpected that IVIg treatment can have modest effects on T cell activation and CD4 count in HIV-1 infection (32, 33). Therefore, it is possible that IVIg could be beneficial as a complement therapy for patients that have residual immune activation despite successful viral control on ART.

## Concluding Remarks

Intravenous immunoglobulin provides CVID patients with a partial replacement for their defective humoral immunity. However, CVID patients also present abnormalities in cellular immunity in a way similar to what is often seen in other conditions associated with chronic immune activation such as HIV-1 infection. Some of these changes are normalized by IVIg treatment, suggesting that IVIg may also be beneficial in other immune deficiencies characterized by persistent immune activation. IVIg seems to have short-lived effects on the monocyte and Treg populations, whereas the reduction of activation in DCs, iNKT cells, and T cells appear to be sustained. However, the vast majority of immunological studies of CVID have been performed on patients who are already on IVIg replacement, therefore, missing or underestimating some of the abnormalities in CVID that are corrected by IVIg. More studies of treatment-naïve CVID patients are needed to better understand what is driving immune activation in CVID and how IVIg is helping to improve cellular immunity. Furthermore, it will be important to investigate how we can restore the compartments that are not recovering after initiation of IVIg, such as Tregs and iNKT cells. Persistent loss of these cells may help explain why some CVID patients still suffer from severe inflammatory complications, such as interstitial lung disease and autoimmune enteropathy, even when on replacement therapy. Cellular therapy has been safely and successfully used to treat two CVID patients suffering from CMV intestinal disease by injection of autologous specific cytotoxic T cell lines expanded *ex vivo* (65). Thus, cellular therapy may represent a good complement to IVIg treatment to help restore a functional immune system in CVID patients.

## Conflict of Interest Statement

The authors declare that the research was conducted in the absence of any commercial or financial relationships that could be construed as a potential conflict of interest.
